# ANXA1 is identified as a key gene associated with high risk and T cell infiltration in primary sclerosing cholangitis

**DOI:** 10.1186/s40246-023-00534-z

**Published:** 2023-09-21

**Authors:** Jian Zhang, Huiwen Wang, Jinqing Liu, Lei Fu, Shifang Peng

**Affiliations:** Department of Infectious Diseases, Xiangya Hospital Central South University, Changsha, China

**Keywords:** ANXA1, Primary sclerosing cholangitis, Immune infiltration, T cell, Prognosis

## Abstract

**Background:**

Primary sclerosing cholangitis (PSC) is a chronic cholestatic liver disease, with unclear pathogenesis. Although immune disorders, especially T cell infiltration, are thought to play a vital role in PSC, the specific pathogenesis mechanisms remain incompletely understood. This study evaluated the potential key gene associated with the PSC pathogenesis and analyzed the associations of the key gene with prognosis and immune cell infiltration by combining bioinformatics analysis and experimental verification.

**Methods:**

Transcriptome data of PSC and normal human liver tissues (GSE159676) were obtained from the gene expression omnibus database. Differentially expressed genes (DEGs) were identified, and differences in biological states were analyzed. A protein–protein interaction (PPI) network was constructed. Hub genes were identified, and their expression was verified using transcriptome data of mice fed 0.1% 3,5-diethoxycarbonyl-1,4-dihydrocollidine (DDC) and *Mdr2*^−/−^ mice (GSE179993, GSE80776), as well as by immunohistochemistry staining on clinical samples. The correlations between the key gene and other factors were evaluated by Pearson’s correlation coefficient. Immune cell infiltration into human liver (GSE159676) was analyzed by xCell and verified by immunofluorescence staining on PSC liver samples.

**Results:**

Of the 185 DEGs identified, 113 were upregulated and 72 were downregulated genes in PSC. Genes associated with immune cell infiltration and fibrosis were significantly enriched in PSC. PPI network showed close interactions among DEGs. A module strongly associated with immune infiltration was identified, with annexin A1 (ANXA1) being the core gene. High expression of ANXA1 in PSC was confirmed in two public datasets and by immunohistochemistry staining on clinical samples. High ANXA1 expression was strongly associated with high-risk score for PSC. Also, ANXA1 expression was positively associated with chemokines and chemokine receptors and with the infiltration of immune cells, especially T cells, into liver with PSC. Immune infiltration, fibrosis, and cancer-related processes were markedly enriched in PSC with high expression of ANXA1.

**Conclusion:**

ANXA1 is a key gene associated with high risk and infiltration of immune cells, especially T cells, in PSC. These findings provide new insight into the key biomarker of PSC and suggest that targeting ANXA1 may be a valuable strategy for the treatment of PSC.

**Supplementary Information:**

The online version contains supplementary material available at 10.1186/s40246-023-00534-z.

## Background

Primary sclerosing cholangitis (PSC) is a chronic cholestatic liver disease characterized by intrahepatic and/or extrahepatic bile duct injury and fibrosis, leading to cholestasis, and biliary cirrhosis with progressive liver dysfunction [1]. The prevalence of PSC has been estimated to 16.2 per 100,000 population, being highest in northern Europe and markedly lower in Asia [2–6]. PSC predominantly affects middle-aged men and is strongly associated with inflammatory bowel disease (IBD) [7]. To date, no effective medical therapy has been developed to cure PSC or slow its progression. In Western countries, PSC represents the leading indication for liver transplantation in patients with cholestatic liver disease, despite the substantial risk of disease recurrence [1, 8]. Better understanding of the molecular pathogenesis of PSC is needed to identify therapeutic targets and develop treatments of this condition.

The pathogenesis and underlying mechanisms of PSC remain incompletely understood. Many hypotheses have been formulated, including genetic susceptibility, autoimmune mechanisms, “leaky gut”, and toxic bile acids [9]. Immune cell infiltration was shown to be strongly associated with cholangiocyte damage and progressive fibrosis [10–13]. T cell infiltration is a hallmark characteristic of PSC, but the composition and functions of these infiltrating T cells have been found to vary [12, 14–16]. Recent studies have emphasized the importance of CD4^+^T cells, CD8^+^ T cells, and regulatory T cells (Tregs) [16–21]. In addition to T cells, several other types of immune cells, including macrophages and neutrophils, have been detected in proximity to the bile ducts of patients with PSC [10, 15]. The exact involvement of each cell type remains unknown. Chemokines and chemokine receptors are factors responsible for the tissue-specific recruitment of these immune cells to sites of inflammation [22]. Although many types of chemokines and chemokine receptors have been found to be abnormally elevated in PSC, resulting in immune cell infiltration [15, 23–26], the key molecules and immune-related regulatory networks have not been determined. Therefore, the identification of disease-associated biomarkers, particularly immune-associated biomarkers, is necessary to better understand the complex pathogenic mechanisms of PSC.

The rapid development of high-throughput technologies provides the opportunity to comprehensively explore the mechanisms underlying rare diseases, including PSC. The Gene Expression Omnibus (GEO) database includes a mass of gene expression profiles of patients with non-neoplastic diseases [27]. This present study was designed to evaluate the potential key genes associated with the PSC pathogenesis and to analyze the associations of key gene with prognosis and immune cell infiltration by analyzing the data in GEO and clinical samples validation. Meanwhile, Gene Ontology (GO) and Kyoto Encyclopedia of Genes and Genomes (KEGG) analyses were also performed to exhibit the differences in biological states between PSC and normal human or between high and low expression of key genes. It is expected to shed light on the identification of the key regulatory factor in PSC.

## Materials and methods

### Data acquisition and processing

The transcriptome datasets GSE159676, GSE179993 and GSE80776 were downloaded from the GEO database. The GSE159676 dataset contained liver tissue samples from 12 patients with PSC and from six healthy control (HC) individuals. The GSE179993 dataset contained liver tissue samples of three mice fed a diet containing 0.1% 3,5-diethoxycarbonyl-1,4-dihydrocollidine (DDC), a model of PSC, and three mice fed normal mouse chow [28, 29]. The GSE80776 dataset contained liver tissue samples from five *Mdr2*^−/−^ mice, another model of PSC, and seven wild-type mice [28, 29].

### Identification of DEGs

DEGs were screened out in all datasets using the limma package in R language, with the cutoffs for significant DEGs defined as an adjusted *p* value < 0.05 and a | log2 fold change (FC) |≥ 1.

### Functional enrichment analysis

Gene Set Enrichment Analysis (GSEA) is a computational method that determines whether an a priori defined set of genes shows statistically significant, concordant differences between two biological states [30, 31]. Differences in biological states between two groups were analyzed using GSEA v4.2.3. Background gene set data required for this study were provided by the Molecular Signatures Database v7.5.1. Functional enrichment analysis was performed using the KEGG C2 subset and the GO C5 subset, with FDR < 25% and nominal *p* < 0.05 considered significantly enriched.

### Establishment of a PPI network and identification of hub genes

Interactive relationships and protein–protein interaction (PPI) networks of DEGs were evaluated using Metascape (http://metascape.org/). The PPI network of key DEGs was visualized using Cytoscape v3.9.1, with hub genes screened by Molecular Complex Detection (MCODE) and cytoHubba in Cytoscape.

### Analysis of immune cell infiltration

xCell is a widely-used gene signature-based method that has been shown to identify 64 types of immune and stromal cells in tissues [32]. Data from the GSE159676 dataset was analyzed by xCell, which quantified the relative proportions of different cells.

### Human liver samples

Paraffin-embedded liver sections, including samples from 6 HC and 7 patients with PSC, were obtained from Xiangya Hospital (Changsha, Hunan, China) between 2017 and 2022. Patients with PSC were diagnosed by experienced physicians based on symptoms, biochemistry, radiology and histological evaluation of biopsied liver tissue. The control liver sections were obtained from normal liver tissues without unusual histological features.

### Immunohistochemistry and immunofluorescence assays

Immunohistochemistry (IHC) staining and immunofluorescence (IF) assays were performed as described [33]. In brief, the paraffin-embedded sections were deparaffinized and hydrated. Antigen retrieval was performed via using Tris–EDTA buffer at pH 9.0. After endogenous peroxidase activity was blocked, the sections were incubated with an anti- annexin A1 (ANXA1) antibody (cat#AF07314, Aifang biological, Changsha, China) at a dilution of 1:200 at 4 °C overnight. Staining was visualized by using 3,3′-diaminobenzidine tetrahydrochloride (cat#SP-9000, ZSBio, Beijing, China) and hematoxylin counterstain. Images were captured using a light microscope (Leica, DMIL LED, Wetzlar, Germany; magnification, × 200). Immunofluorescence staining was performed by the incubation of samples with an anti-ANXA1 antibody (cat#AF07314, Aifang biological, Changsha, China) at a dilution of 1:100 and an anti-CD3 antibody (cat# sc-20047, Santa Cruz Biotechnology, Dallas, US) at a dilution of 1:50 at 4 ◦C overnight, followed by incubation with appropriate secondary antibodies at 37 ◦C for 1 h. Then, DAPI (Boster Biological Technology, Wuhan, China) was used for nuclear staining performed 5 min. Fluorescent signals were captured and merged using a laser scanning confocal microscope (ZEISS LSM880; Oberkochen, Germany). At 20x, three fields of view were selected for each sample for statistical analysis. Using Image-pro plus 6.0 to quantity the percentage of stained areas.

### Statistical analysis

Data were represented as mean ± standard deviation (SD). All data were statistically analyzed using Graphpad Prism v9.1.1. Correlations between two factors were evaluated by Pearson’s correlation analysis. Significance of differences between two groups was determined by two-tailed independent-sample Student's t test (Mann–Whitney U test for data showing skewed distribution) and *p* value < 0.05 was considered statistically significant.

## Results

### Identification of DEGs in PSC

**Fig. 1:**
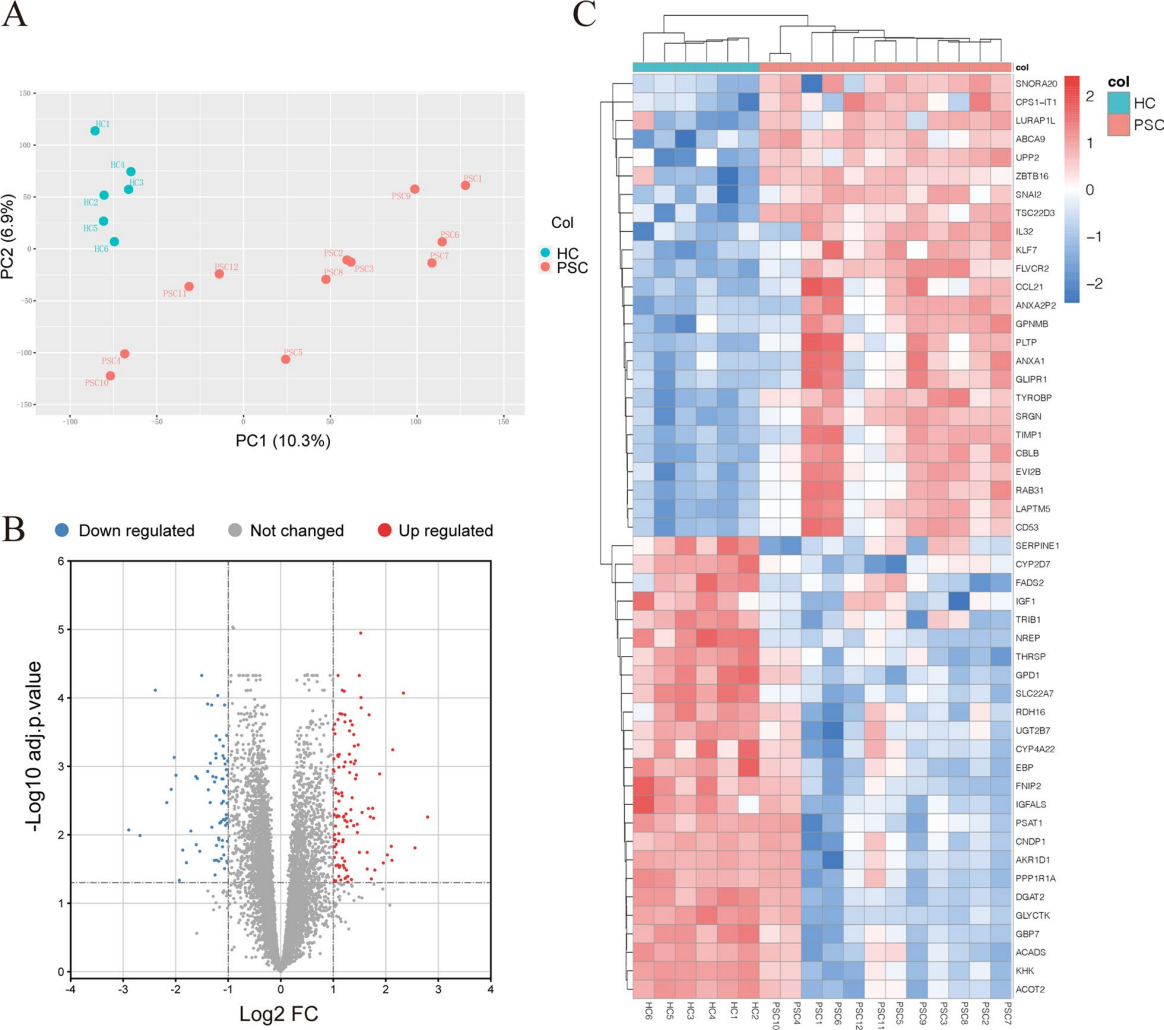
Identification of DEGs between PSC and HC in the GSE159676 dataset. **A** PCA map of GSE159676. **B** Volcano map of all DEGs analyzed by the limma package in R language; an adjusted *p* value < 0.05 and a | log2 FC |≥ 1 were considered threshold values for significant DEGs. **C** Heatmap of the top 50 DEGs, including the top 25 upregulated and the top 25 downregulated genes in PSC. DEGs, differentially expressed genes; PSC, primary sclerosing cholangitis; HC, healthy control; PCA, principal component analysis

The microarray dataset GSE159676 obtained from the GEO database included 18 liver tissue samples, 12 from patients with PSC and six from HC. Principal component analysis (PCA) analysis showed that PSC and HC could be well distinguished (Fig. 1A). Analysis using the limma package and the criteria |logFC|≥ 1 and adjusted *p* value < 0.05, identified 113 DEGs as being upregulated and 72 as downregulated in PSC samples (Additional file 1). A volcano map of DEGs (Fig. 1B) and a heatmap of the top 50 DEGs (Fig. 1C) showed significant differences in gene expression between PSC and HC.

### Functional enrichment analysis of PSC

**Fig. 2:**
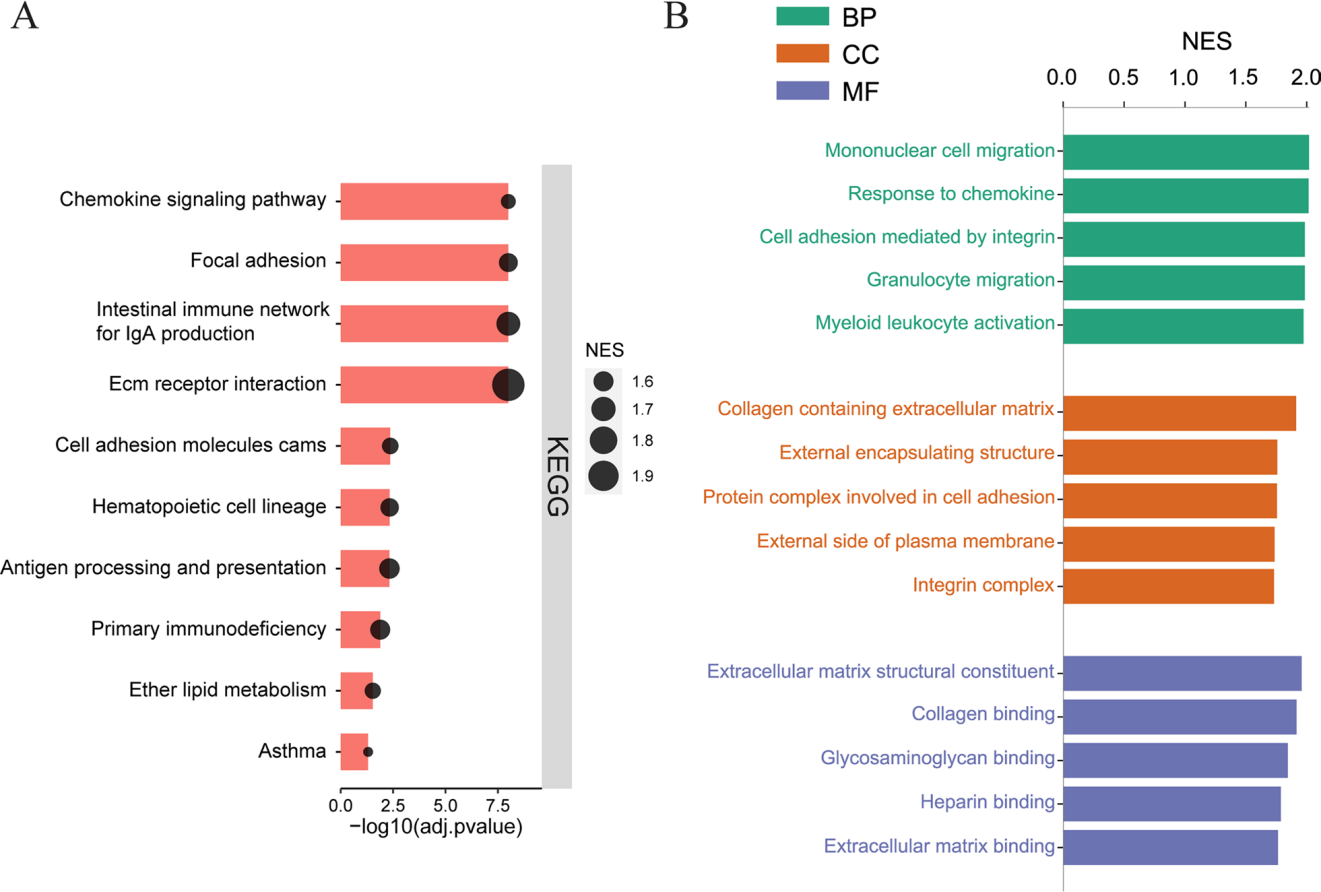
Functional enrichment analysis by GSEA showing differences in biological states between PSC and HC. **A** Top 10 KEGG pathways in PSC. **B** Top 5 GO-BP, GO-CC and GO-MF in PSC. GSEA, gene set enrichment analysis; PSC, primary sclerosing cholangitis; HC, healthy control; KEGG, kyoto encyclopedia of genes and genomes; GO, gene ontology; BP, biological process; CC, cellular components; MF, molecular function

Overall differences in biological states between PSC and HC were evaluated by GSEA. KEGG pathway analysis showed that the top 10 pathways enriched in the PSC samples included those involved in extracellular matrix (ECM) receptor interactions, immune-associated pathways or diseases, focal adhesion, hematopoietic cell lineage, cell adhesion molecules, lipid metabolism, and chemokine signaling pathways (Fig. 2A). Most of these pathways were closely associated with the adhesion, migration, activation, differentiation and proliferation of immune cells. Similar results in GO analysis, biological processes (BPs) included the migration, activation and interaction of immune cells (Fig. 2B). Cellular components (CCs) and molecular functions (MFs) analyses also indicated that the ECM and collagen, both of which were associated with fibrosis, were functions enriched in PSC. These findings were consistent with previous results [9, 13], indicating that immune infiltration and fibrosis are important pathological features of PSC. In particular, the abnormal activation and migration of various immune system cells provided evidence for the importance of immune dysfunction in PSC.

### Establishment of a PPI network and identification of hub genes

**Fig. 3:**
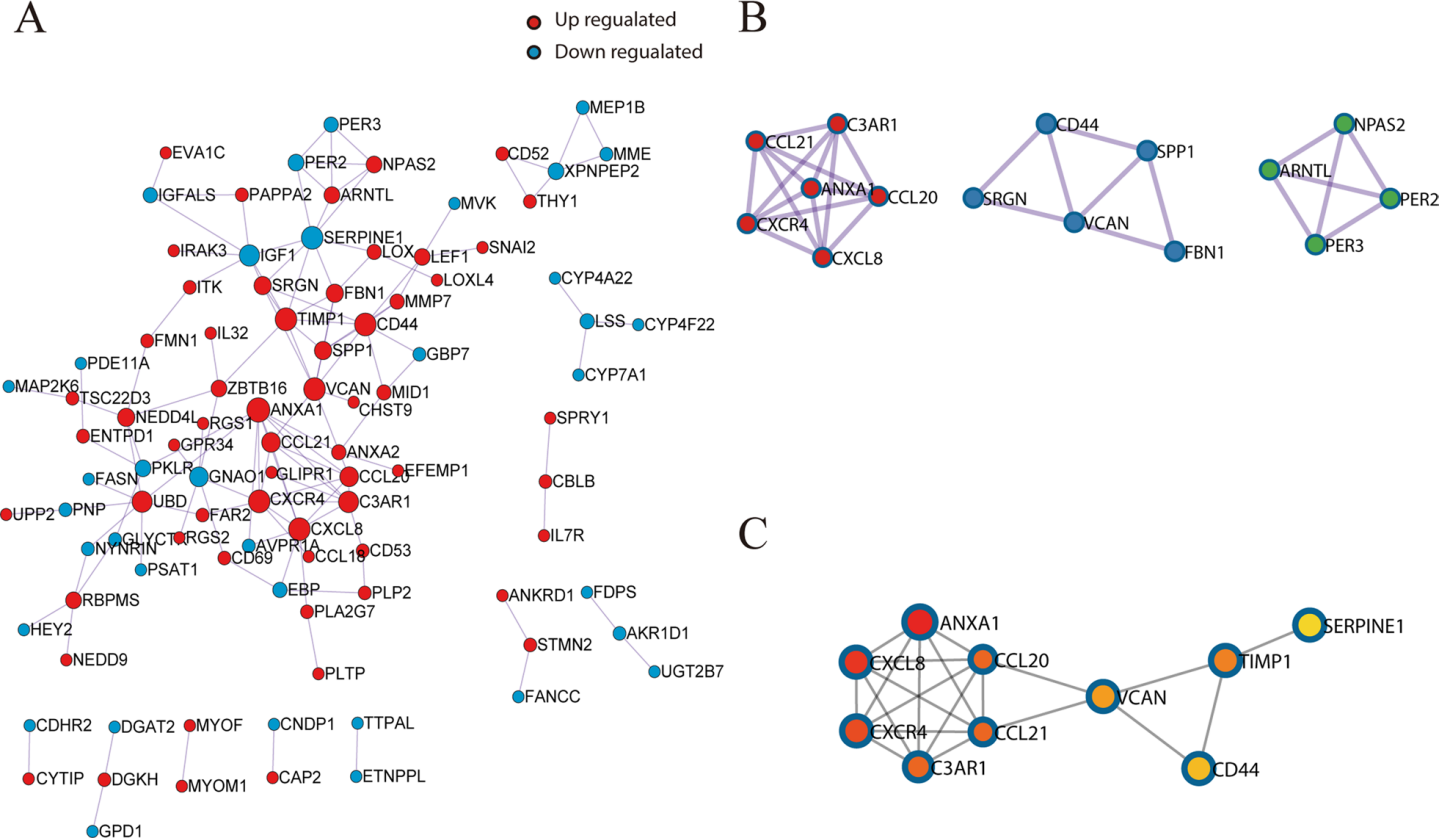
PPI network of DEGs constructed by Metascape and identification of hub genes **A** Construction of a PPI network of DEGs, consisting of 96 nodes and 129 edges, using Metascape. **B** The three modules with the highest scores constructed by MCODE in Metascape. **C** The top10 hub genes constructed by Cytohubba using the MCC method. PPI, protein–protein interaction; DEGs, differentially expressed genes; MCODE, molecular complex detection; MCC, maximal clique centrality

A stable PPI network of DEGs was constructed using Metascape and visualized by Cytoscape. The PPI network consisted of 96 nodes and 129 edges (Fig. 3A). Hub genes were identified by combining MCODE, which is supported by Metascape, and cytoHubba, an App in Cytoscape. MCODE identified three modules (Fig. 3B), whereas the maximal clique centrality (MCC) method in cytoHubba selected the top 10 hub genes. The common Hub genes in the two groups included ANXA1, CXCL8, CCL21, CCL20, CXCR4, C3AR1, VCAN, and CD44 (Fig. 3B, C), with one module of hub genes, consisting of ANXA1, CXCL8, CCL21, CCL20, CXCR4, and C3AR1, present in both results. This module was closely associated with immune infiltration, with ANXA1, the core gene of this module, having the strongest interaction with other genes. These findings suggested that ANXA1 potentially plays a crucial role in PSC.

### Validation of ANXA1 expression level and correlation with prognosis

**Fig. 4:**
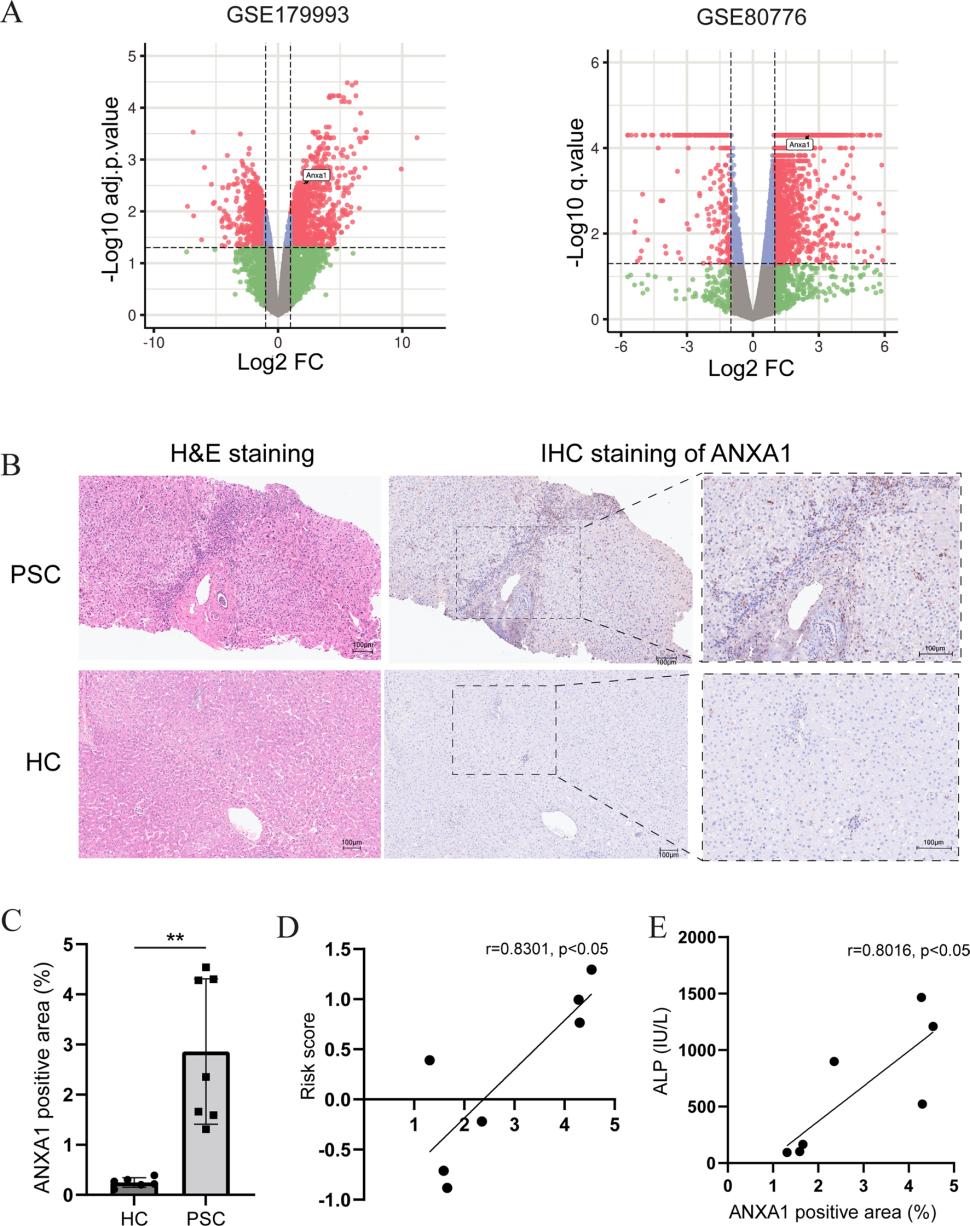
Validation of ANXA1 expression level and correlation with prognosis. **A** Volcano map of the GSE179993 dataset, containing the transcriptome data of liver tissue samples from three 0.1% DDC-fed mice and three mice fed normal chow, and showing the expression of ANXA1. Volcano map of the GSE80776 dataset, containing the transcriptome data of liver tissue samples of five *Mdr2*^−/−^ and seven wild-type mice, and showing the expression of ANXA1. **B** Representative IHC and H&E staining images from serial liver sections in PSC and HC. **C** Quantification of ANXA1 expression in PSC and HC by analyzing the stained areas of IHC images. **D** The correlations between ANXA1 expression and risk score by using the revised natural history model for PSC, **E** and serum ALP. **p < 0.01. ANXA1, annexin A1; IHC, immunohistochemistry; H&E, hematoxylin and eosin staining; PSC, primary sclerosing cholangitis; HC, healthy control; ALP, alkaline phosphatase;

The involvement of ANXA1 expression in PSC was evaluated in two mouse models of this disease, mice fed a diet containing 0.1% DDC and *Mdr2*^−/−^ mice [28, 29]. Two datasets were obtained from the GEO database, the GSE179993 and GSE80776 datasets. The GSE179993 dataset contained liver tissue samples from three mice fed 0.1% DDC and three mice fed normal mouse chow, whereas the GSE80776 dataset contained liver tissue samples from five *Mdr2*^−/−^ and seven wild-type mice. Volcano maps showed that the levels of expression of ANXA1 were significantly higher in mice fed 0.1% DDC and in *Mdr2*^−/−^ mice than in their respective controls (Fig. 4A).

To further verify the ANXA1 expression, we performed immunohistochemical staining on 7 PSC and 6 healthy liver samples. The clinical features of patients were showed in Table 1. ANXA1 was barely expressed in healthy liver, but significantly elevated in PSC (Fig. 4C), especially in the portal area (Fig. 4B). The revised natural history model for PSC is the widely used prognostic assessment method and can be used to obtain survival estimates up to 4 years of follow-up [34]. According to the linear correlation analysis, the expression of ANXA1 showed a significant positive correlation with risk score in PSC (Fig. 4D). Besides, it also exhibited a strong association with serum alkaline phosphatase (ALP) (Fig. 4E), which is another biochemical indicator correlated with the severity of PSC [35, 36]. All these results provided further evidence that elevated ANXA1 levels in PSC might lead to a poor prognosis.

**Table 1:** Clinical features of healthy controls and PSC patients

Clinical features	Healthy controls	PSC patients
Total samples (male/female)	6(2/4)	7(2/5)
Age (years)	49.83 ± 5.42	33.29 ± 20.41
ALT (IU/L)	12.02 ± 3.49	82.96 ± 106.10
AST (IU/L)	17.67 ± 2.60	159.2 ± 284.00*
ALB (g/L)	36.32 ± 4.83	37.64 ± 5.41
ALP (IU/L)	82.63 ± 10.30	636.80 ± 562.50**
GGT (IU/L)	25.48 ± 15.09	286.20 ± 247.60
TBA (μmol/L)	5.75 ± 4.44	32.79 ± 46.00
TBIL (μmol/L)	8.95 ± 2.67	45.31 ± 59.27*
DBIL (μmol/L)	3.32 ± 1.76	23.80 ± 28.99**
History of variceal bleeding	0	0

Values are means ± SD. *p < 0.05, **p < 0.01

*PSC* primary sclerosing cholangitis; *ALT* alanine aminotransferase; *AST* aspartate aminotransferase; *ALB* albumin; *ALP* alkaline phosphatase; *GGT* gamma-glutamyl transferase; *TBA* total bile salts; *TBIL* total bilirubin; *DBIL* direct bilirubin

### Relationship between ANXA1 and chemokines or chemokine receptors

**Fig. 5:**
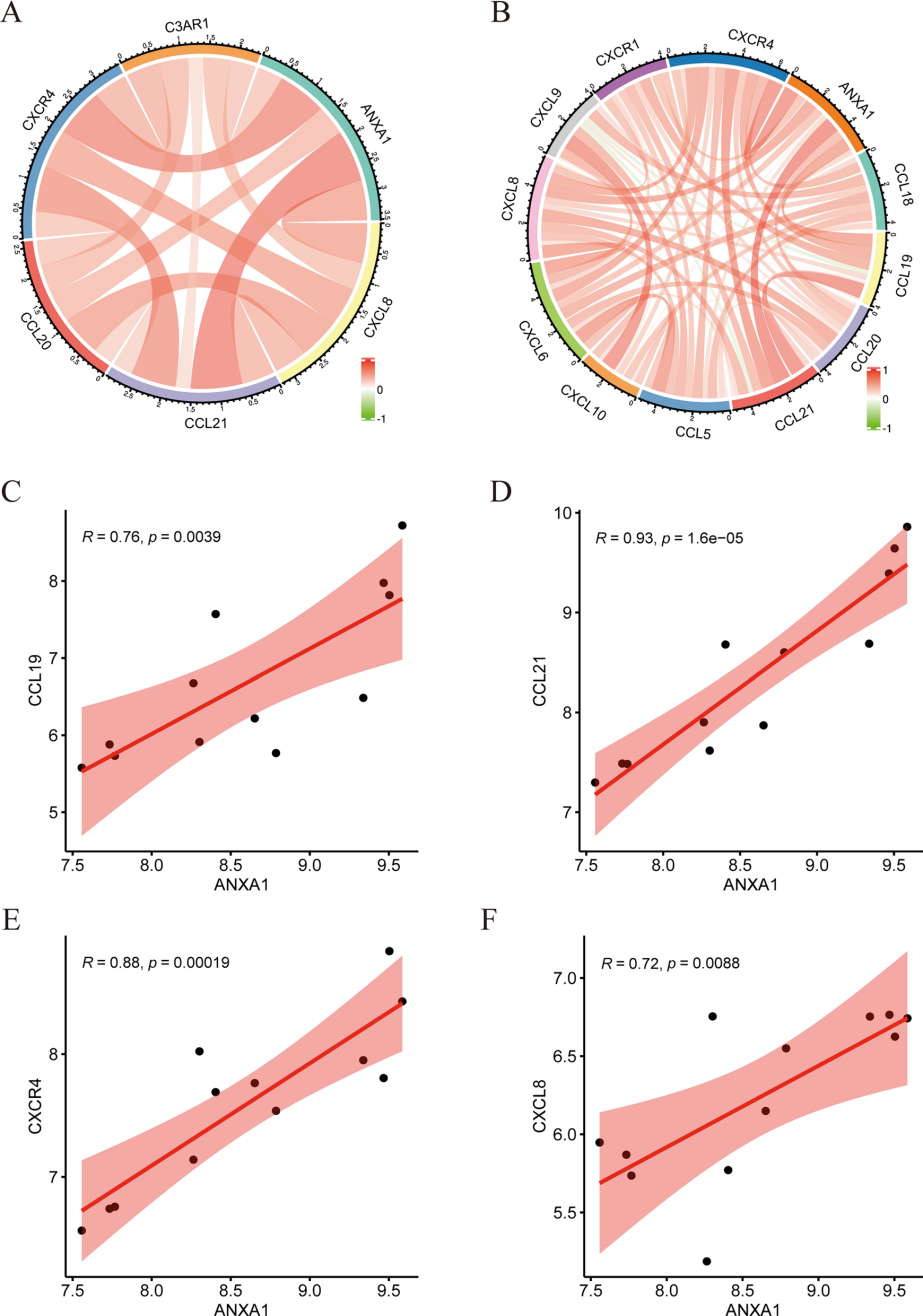
Relationships of ANXA1 expression with the expression of chemokines and chemokine receptors. **A** Positive correlations between ANXA1 expression and the expression of other hub genes in the module, all of which encode pro-inflammatory factors. **B** Positive correlations between ANXA1 and the expression of additional chemokines and chemokine receptors when DEGs were relaxed to FC ≥ 1.5. **C**–**F** Statistically significant correlations of ANXA1 expression with the expression of chemokines and chemokine receptors, including **C** CCL19, **D** CCL21, **E** CXCR4, and **F** CXCL8. ANXA1, annexin A1; DEGs, differentially expressed genes; CCL, C-C motif chemokine ligand; CXCR, C-X-C motif chemokine receptor; CXCL, C-X-C motif chemokine ligand

The function of ANXA1 in PSC was initially evaluated by assessing the hub genes that strongly interacted with ANXA1 in above-described module. Of the five genes identified, four, CXCL8, CCL21, CCL20, and CXCR4 encode chemokines or chemokine receptors, which promote and direct immune cell migration. CXCL8 mainly recruits neutrophils, CCL21 and CCL20 mainly recruit T cells, and CXCR4 is a chemokine receptor mainly expressed in T cells [22]. The fifth gene, C3AR1 encodes a C3a receptor. C3a is a peptide that enhances effector T cell proliferation and survival, while simultaneously inhibiting the induction and function of Tregs, thereby exacerbating inflammation [37]. The relationships between ANXA1 and these pro-inflammatory factors were further explored by Pearson’s correlation analysis, which found that ANXA1 expression correlated positively with all five of these pro-inflammatory factors (Fig. 5A). Due to the importance of chemokines in promoting inflammation, the relationships between ANXA1 and more highly expressed chemokines and chemokine receptors were evaluated. Relaxing the restriction of DEGs to FC ≥ 1.5 showed that CXCL6, CXCL9, CXCL10, CCL5, CCL18, CCL19, and CXCR1 interacted with ANXA1, with Pearson’s correlation analysis showing that the expression of ANXA1 correlated positively with the levels of expression of these chemokines and chemokine receptors (Fig. 5B). ANXA1 expression correlated significantly with the levels of CCL19, CCL21, CXCL8, and CXCR4 (Fig. 5C–F), with three of these, CCL19, CCL21, and CXCR4, mainly acting on T cells. Taken together, these results indicate that ANXA1 may act as a positive regulator of many chemokines and chemokine receptors, especially those related to T cells.

### Functional enrichment analysis of PSC with high ANXA1 expression levels

**Fig. 6:**
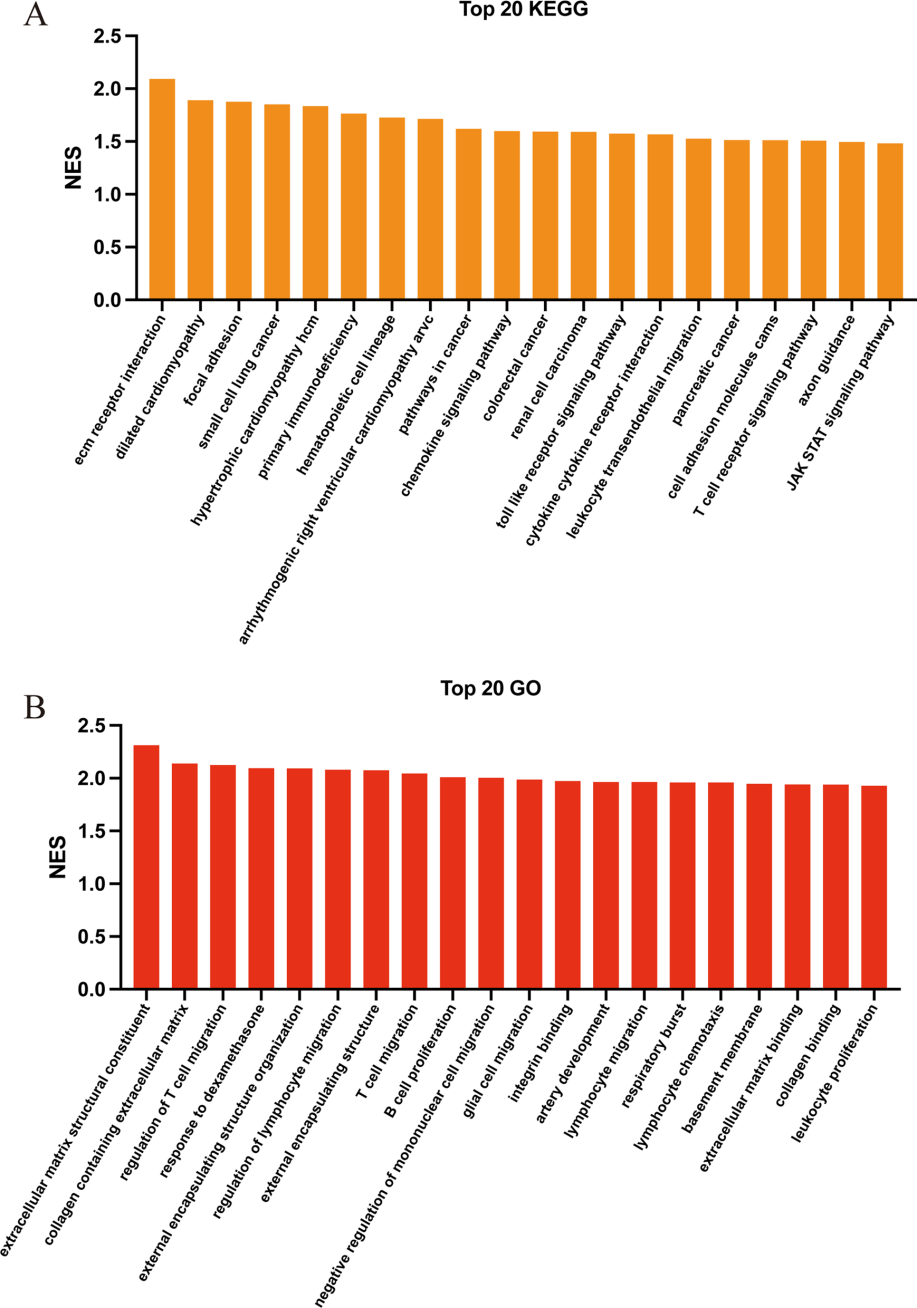
Functional enrichment analysis of PSC with high ANXA1 expression level. Samples were divided into those with high (n = 6) and low (n = 6) ANXA1 expression based on the median concentration. **A** Top 20 KEGG pathways in the high ANXA1 group. **B** Top 20 GO pathways following integration of GO-BP, GO-CC, and GO-MF, in the high ANXA1 group. PSC, primary sclerosing cholangitis; ANXA1, annexin A1; KEGG, kyoto encyclopedia of genes and genomes; GO, gene ontology; BP, biological process; CC, cellular components; MF, molecular function; NES, normalized enrichment score

To further assess the effects of ANXA1 expression on PSC, 12 samples of PSC in the GSE159676 dataset were divided into those with low (6 samples) and high (6 samples) ANXA1 expression based on the median level of ANXA1 expression. GSEA was utilized in enrichment analysis of the two groups. The top 20 KEGG pathways in the high ANXA1 group included several cell adhesion and immune associated pathway, such as those involved in ECM receptor interaction, focal adhesion, primary immunodeficiency, hematopoietic cell lineage, toll like receptor signaling, cytokine-receptor interactions, leukocyte transendothelial migration, cell adhesion molecules, T cell receptor signaling and JAK-STAT signaling. Interestingly, several cancer-related pathways were also enriched in the high ANXA1 group (Fig. 6A). The results of Top 20 GO enrichment analysis (including GO-BP, GO-CC, and GO-MF) were similar, with almost all of these biological states being related to immune infiltration and fibrosis. Importantly, many lymphocytes related processes were involved, especially those associated with T cells, including regulation of T cell and lymphocyte migration, and lymphocyte chemotaxis (Fig. 6B). These findings, together with results showing that ANXA1 expression correlated positively with the expression of T cell associated chemokines and chemokine receptors, suggested that ANXA1 was closely related to T cell infiltration.

### Relationship between ANXA1 expression and T cell infiltration in PSC livers

**Fig. 7:**
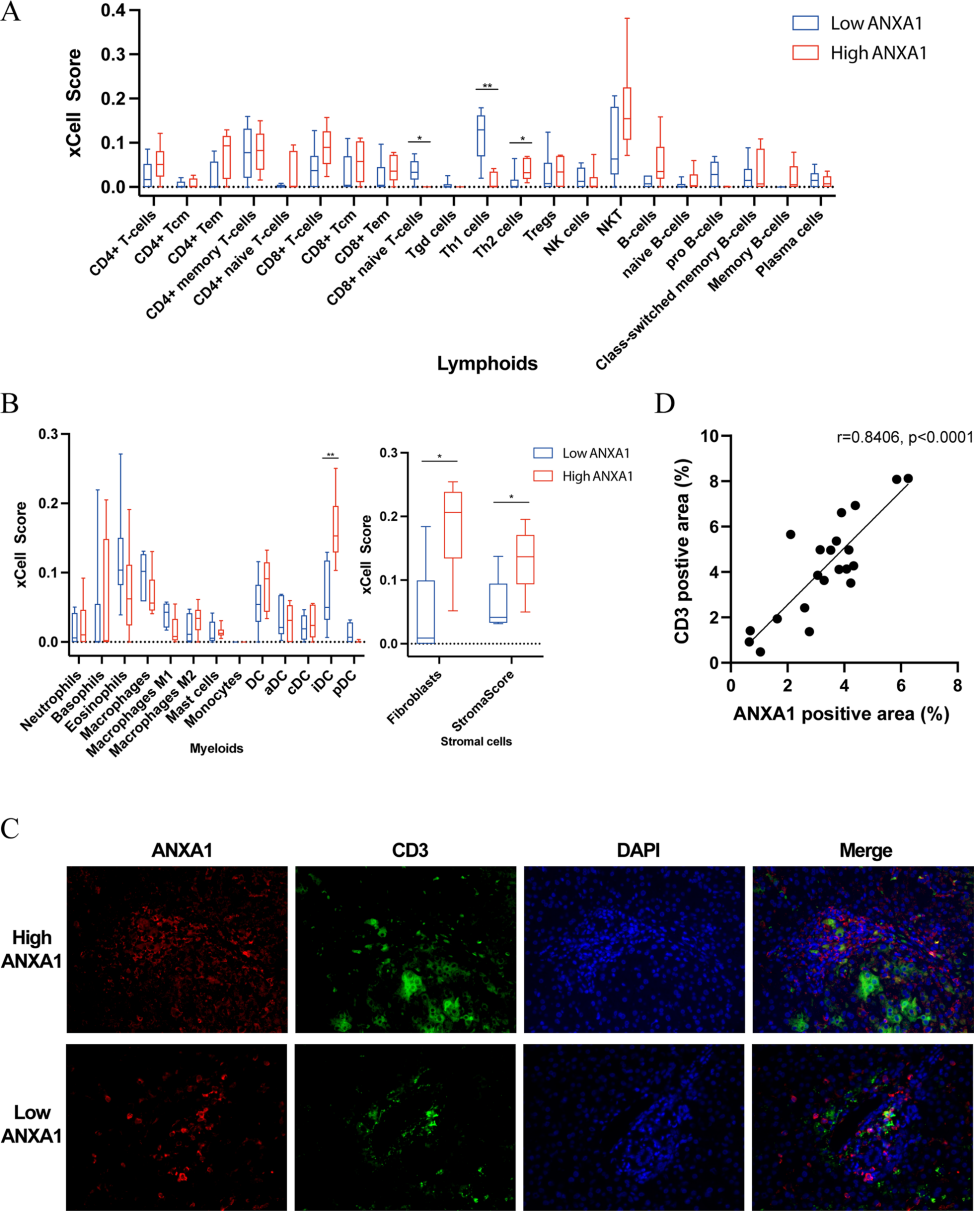
Relationship between ANXA1 expression and immune cell infiltration in PSC livers, as shown by xCell analysis of the GSE159676 dataset. Infiltration of **A** lymphoid cells, **B** myeloid cells and stromal cells into PSC livers in the low and high ANXA1 groups. **C** Representative IF staining images for ANXA1 and CD3 in PSC patients’ liver samples. **D** The correlation between ANXA1 positive area and CD3 positive area in IF staining images from PSC patients. *p < 0.05, **p < 0.01, ***p < 0.001. ANXA1, annexin A1; PSC, primary sclerosing cholangitis; IF, immunofluorescence

To further assess the correlation of ANXA1 expression with immune cell infiltration, the density of immune cells was compared in the livers of the high and low ANXA1 groups using xCell. Infiltration of most lymphocytes was greater in the high than in the low ANXA1 group (Fig. 7A). Statistically significant differences were observed in CD8^+^ naive T cells, Th1 cells, and Th2 cells, with CD8^+^ naive T cells and Th1 cells being significantly lower and Th2 cells significantly higher in the high ANXA1 group. In addition, populations of dendritic cells (DCs), which have been linked to activation of T cells, were higher in the high ANXA1 group, with the infiltration of iDCs being significantly higher in the high ANXA1 group (Fig. 7B). Because high expression of ANXA1 may also be associated with fibrosis, the proportions of stromal cells were also analyzed by xCell. Fibroblast populations and StromaScore were higher in the high ANXA1 group, suggesting that ANXA1 may also be associated with liver fibrosis in patients with PSC (Fig. 7B).

To identify the predicted relationship between ANXA1 and T cell, we performed IF staining on PSC patients’ liver samples. It seemed that ANXA1 was not predominantly expressed on T cell, but region with high ANXA1 expression was accompanied by more CD3^+^ T cell infiltration too (Fig. 7C). Furthermore, we randomly selected three fields of view for each sample, counted the percentage of ANXA1 and CD3 positive regions, and performed a Pearson’s correlation analysis. The result suggested ANXA1 expression correlated significantly with the levels of CD3^+^ T cell (Fig. 7D). These findings provided further indication that ANXA1 was positively related to T cell infiltration, in the liver of patients with PSC.

## Discussion

PSC is a complex liver disease of as yet incompletely understood pathogenesis. Immune dysfunction, especially T cell dysfunction, has been shown to play an important role in the pathogenesis of PSC, but specific regulatory mechanisms remain unclear. Understanding of PSC is limited by its relatively low incidence and lack of pathological samples. High throughput technologies allow further evaluation of the mechanisms underlying the pathogenesis of PSC. The present study used several methods of bioinformatics analysis to evaluate the transcriptome data of PSC. These analyses showed that ANXA1 was a potential key gene correlated with infiltration of immune cells, especially T cells, in PSC. These findings provide further insight into the mechanisms underlying the immune dysfunction of PSC, suggesting that ANXA1 may be a target for therapeutic intervention in PSC.

Several types of immune cells, especially T cells, have been shown to be increased in the PSC livers [15]. Similar results were observed in the present study, with enrichment analysis indicating that several biological pathways, including those involved in the adhesion, migration, activation, differentiation and proliferation of immune cells, were up-regulated in PSC (Fig. 2). Bioinformatics analysis of 185 DEGs revealed a module of hub genes, most of which had pro-inflammatory activities (Fig. 3). These results provided further evidence for the importance of immune dysfunction in PSC.

ANXA1 was the core gene in the module. To confirm the involvement of ANXA1 in PSC, ANXA1 expression was assessed in other two datasets and in patients. ANXA1 was found to be overexpressed in human PSC and in animal models (Fig. 4), and to be positively correlated with the expression of several chemokines and chemokine receptors (Fig. 5). Enrichment analysis showed that immune cell infiltration, especially T cell infiltration, was higher in PSC with high expression of ANXA1 than that with low expression of ANAX1. The positive relationship between ANXA1 expression and T cell infiltration into the liver of patients with PSC was also confirmed by xCell analysis and IF staining (Fig. 7). Taken together, these findings illustrated that ANXA1 was closely associated with immune cell infiltration in patients with PSC.

ANXA1 belongs to the annexin superfamily of calcium-dependent phospholipid-binding proteins and is regulated by glucocorticoids to inhibit the action of cytosolic phospholipase A2 (PLA2) [38–40]. This, in turn blocks the release of arachidonic acid, preventing the synthesis of eicosanoids [38–40]. ANXA1 is regarded as an anti-inflammatory factor in the innate immune system, as it inhibits neutrophil accumulation and promotes the clearance of apoptotic leukocytes by macrophages [41, 42]. In adaptive immunity, however, ANXA1 was reported to enhance the proliferation and activation of T cells. The addition of human recombinant ANXA1 (hrANXA1) to T cells stimulated with CD3 and CD28 was found to enhance T cell proliferation and activation [43, 44]. Rheumatoid arthritis (RA) is an autoimmune disease with many similarities to PSC. Both diseases are associated with genetic susceptibility and T cell dominant infiltration [45]. Interestingly, the expression of ANXA1 in T cells was found to be higher in patients with RA than in healthy control volunteers [43]. Furthermore, administration of hrANXA1 during the immunization phase in an animal model of RA (collagen-induced arthritis) was found to further enhance the signs and symptoms of arthritis [43]. These findings, as well as the present results, indicate that ANXA1 may play a significant role in PSC by activating and promoting T cells. In contrast to other autoimmune diseases, glucocorticoids are ineffective against PSC [46, 47]. The results of the present study may also partly explain this ineffectiveness. Because ANXA1 is the main effector molecule of glucocorticoids, glucocorticoids may induce the production of large amounts of ANXA1, which, in most autoimmune diseases, inhibits inflammation. Because ANXA1 could promote T cell infiltration in PSC, treatment with glucocorticoids may enhance T cell proliferation. Notably, our study found that ANXA1-positive cells were predominantly accumulated in the portal area of livers from PSC patients, but only few of CD3^+^ T cells expressed ANXA1 (Fig. 7C). This suggests that T cells are not the main source of ANXA1 in the liver of PSC patients. Previous publications have shown that ANXA1 is widely expressed in leukocytes, lymphocytes, and epithelial cells [48]. Therefore, the exactly cellular sources of ANXA1 and the underlying mechanisms in PSC still need to be further investigated in the future studies.

Recent studies have revealed that genetic susceptibility has a key role in the pathogenesis of PSC [49]. Several relevant loci associated with PSC have now been identified through genome-wide association studies (GWAS), including human leukocyte antigens (HLA)-associated as well as non-HLA-associated loci [49–51]. HLA susceptibility variants usually are considered to be closely related to immune function [52]. So far, a lot of HLA-associated risk loci have been confirmed in PSC, such as the DQA*01:03, DQA*01:03, DQA*05:01, DRB1*15:01, DQA*01:01[53]. Interestingly, one study showed that ANXA1 acts as an antigen to raise both antibody production and T cell response by interacting with HLA [54]. However, it remains to be explored whether ANXA1 has the same function and whether it could be affected by HLA variants in PSC. In addition, a growing number of research have found that many non-HLA -associated loci are also highly associated with PSC, especially these genes associated with T cell function, such as TNFRSF14, CD28, IL2 / IL21, and IL2RA [49, 50, 55]. Notably, it has been demonstrated that ANXA1 promotes CD28/IL2-mediated T cell proliferation [43, 56]. Furthermore, a recent study identified a series of new susceptibility loci to PSC and subsequent PPI analysis showed that these candidate genes were linked to several immune response pathways, such as T cell receptor, cytokine, chemokine, JAK-STAT, and toll-like receptor signaling pathways [50]. This finding fits well with our results that the high ANXA1group were also enriched with these biological pathways compared to the low ANXA1 group (Fig. 6). Thus, we hypothesized that ANXA1 may be involved in these pathogenic processes related to both HLA-associated and non-HLA-associated susceptibility loci.

Several cancer related pathways were found to be enriched in the high ANXA1 group (Fig. 6). ANXA1 expression was found to vary, depending on tumor type, suggesting it may play a role in the regulation of tumor cell proliferation and tumor growth [48]. ANXA1 was recently reported to be a marker for cholangiocarcinoma [57, 58]. Although these findings were consistent with results showing that PSC may progress to cholangiocarcinoma, the role of ANXA1 in cholangiocarcinoma remains to be determined.

This study had several limitations. We were unable to obtain enough blood samples of PSC patients to analyze the possibility of serum ANXA1 as a diagnostic biomarker. In addition, we could not further obtain more clinical samples to verify the association of ANXA1 with different kinds of T cells. Nonetheless, the findings of this study were likely reliable, as they were based on multiple datasets from both humans and mice, as well as experimental verification in human liver samples. In addition, findings showing the relationships of ANXA1 with chemokines and immune cell infiltration, as well as the results of enrichment analysis of PSC with high ANXA1 expression, clearly indicate that ANXA1 plays a vital role in promoting T cell infiltration in PSC. Further studies are needed to evaluate the mechanisms by which ANXA1 regulates T cells in PSC and to verify whether targeting ANXA1 can effectively treat PSC.

## Conclusion

Using bioinformatics analyses and experimental verification, the present study showed that ANXA1 is a key gene correlated with prognosis and T cell infiltration in PSC. It suggests that ANXA1 may be a promising therapeutic target in this disease.

### Supplementary Information

**Additional file 1**. Details of 185 DEGs.

## Data Availability

The datasets GSE159676, GSE179993, and GSE80776 for this study can be found in the GEO (http://www.ncbi.nlm.nih.gov/geo). Further inquiries can be directed to the corresponding authors.

## Abbreviations

ANXA1: Annexin A1
PSC: Primary sclerosing cholangitis
GEO: Gene expression omnibus
HC: Healthy control
DEGs: Differentially expressed genes
PPI: Protein–protein interaction
DDC: 3,5-Diethoxycarbonyl-1,4-dihydrocollidine
GO: Gene ontology
BP: Biological processes
CC: Cellular components
MF: Molecular functions
NES: Normalized enrichment score
KEGG: Kyoto encyclopedia of genes and genomes
GSEA: Gene set enrichment analysis
FC: Fold change
FDR: False discovery rate
GAPDH: Glyceraldehyde-3-phosphate dehydrogenase
PCA: Principal components analysis
ECM: Extracellular matrix
ALT: Alanine aminotransferase
AST: Aspartate aminotransferase
ALB: Albumin
ALP: Alkaline phosphatase
GGT: Gamma-glutamyl transferase
TBA: Total bile salts
TBIL: Total bilirubin
DBIL: Direct bilirubin
CXCL8: C-X-C motif chemokine ligand 8
CCL21: C-C motif chemokine ligand 21
CCL20: C-C motif chemokine ligand 20
CXCR4: C-X-C motif chemokine receptor 4
C3AR1: Complement C3a receptor 1
VCAN: Versican
CXCL6: C-X-C motif chemokine ligand 6
CXCL9: C-X-C motif chemokine ligand 9
CXCL10: C-X-C motif chemokine ligand 10
CCL5: C-C motif chemokine ligand 5
CCL18: C-C motif chemokine ligand 18
CCL19: C-C motif chemokine ligand 19
CXCR1: C-X-C motif chemokine receptor 1
Tregs: Regulatory T cells
DCs: Dendritic cells
iDCs: Immature dendritic cells
aDCs: Activated dendritic cells
cDCs: Conventional dendritic cells
pDCs: Plasmacytoid dendritic cells
PLA2: Phospholipase A2
RA: Rheumatoid arthritis
GWAS: Genome-wide association studies
HLA: Human leukocyte antigens
