# Supplementary material for: ANXA1 is identified as a key gene associated with high risk and T cell infiltration in primary sclerosing cholangitis
Source: Hum Genomics. 2023 Sep 21;17:86. doi: 10.1186/s40246-023-00534-z (PMC10512524; doi:10.1186/s40246-023-00534-z)
Supplement: Supplementary file 1 — Additional file 1. Details of 185 DEGs. [file 40246_2023_534_MOESM1_ESM.docx]

Additional file 1

# Supplementary Table 1. Details of 185 DEGs

| **Symbols** | **EntrezID** | **logFC** | **adj.P.Val** |
| --- | --- | --- | --- |
| CYP7A1 | 1581 | -2.8937 | 0.008495 |
| BBOX1 | 8424 | -2.6786 | 0.010246 |
| NCAM2 | 4685 | -2.3868 | 7.72E-05 |
| THRSP | 7069 | -2.1701 | 0.003383 |
| SLC5A12 | 159963 | -2.0847 | 0.00217 |
| MIR21 | 406991 | -2.0276 | 0.000741 |
| MEP1B | 4225 | -1.9942 | 0.001351 |
| MME | 4311 | -1.9296 | 0.046245 |
| FREM2 | 341640 | -1.8659 | 0.01676 |
| XPNPEP2 | 7512 | -1.7951 | 0.025578 |
| BCHE | 590 | -1.7119 | 0.008794 |
| CDHR2 | 54825 | -1.6169 | 0.001407 |
| AVPR1A | 552 | -1.6075 | 0.013951 |
| DGAT2 | 84649 | -1.587 | 0.001503 |
| SERPINE1 | 5054 | -1.5411 | 0.017418 |
| NREP | 9315 | -1.5046 | 4.70E-05 |
| GPD1 | 2819 | -1.3905 | 0.001186 |
| SLC22A7 | 10864 | -1.3904 | 0.000123 |
| GNAO1 | 2775 | -1.3793 | 0.002201 |
| CYP4F22 | 126410 | -1.3424 | 0.003384 |
| PDE11A | 50940 | -1.34 | 0.000901 |
| CACNA1H | 8912 | -1.3149 | 0.000127 |
| PER3 | 8863 | -1.3119 | 0.007758 |
| HEPACAM | 220296 | -1.2922 | 0.001407 |
| PSAT1 | 29968 | -1.264 | 0.023684 |
| PER2 | 8864 | -1.2599 | 0.001692 |
| CNDP1 | 84735 | -1.2501 | 0.038663 |
| OSBPL6 | 114880 | -1.2367 | 0.000655 |
| LSS | 4047 | -1.2328 | 0.000756 |
| KHK | 3795 | -1.231 | 0.001452 |
| NECAB2 | 54550 | -1.2308 | 0.000358 |
| FADS2 | 9415 | -1.2245 | 0.023486 |
| PPP1R1A | 5502 | -1.2234 | 0.014322 |
| HEY2 | 23493 | -1.1985 | 9.24E-05 |
| VNN3 | 55350 | -1.1809 | 0.011136 |
| ETNPPL | 64850 | -1.1807 | 0.024307 |
| FASN | 2194 | -1.1759 | 0.011814 |
| ISM1 | 140862 | -1.1611 | 0.000409 |
| TRIB1 | 10221 | -1.1594 | 0.006747 |
| GFRA1 | 2674 | -1.149 | 0.006638 |
| AKR1D1 | 6718 | -1.1311 | 0.022311 |
| ACADS | 35 | -1.1303 | 0.006407 |
| ACOT2 | 10965 | -1.1241 | 0.003469 |
| PNP | 4860 | -1.12 | 0.001528 |
| GSTA3 | 2940 | -1.1098 | 0.01209 |
| UGT2B7 | 7364 | -1.1089 | 0.014977 |
| MIR107 | 406901 | -1.1001 | 0.0007 |
| TTPAL | 79183 | -1.0992 | 0.001527 |
| MVK | 4598 | -1.0949 | 0.000506 |
| FNIP2 | 57600 | -1.0929 | 0.003243 |
| CYP2D7 | 1564 | -1.0923 | 0.005971 |
| DEPDC7 | 91614 | -1.0875 | 0.002289 |
| STARD4 | 134429 | -1.0819 | 0.019704 |
| ATP11C | 286410 | -1.076 | 0.000765 |
| SYT7 | 9066 | -1.0712 | 0.00243 |
| IGF1 | 3479 | -1.0693 | 0.031183 |
| TBX15 | 6913 | -1.0691 | 0.000127 |
| PKLR | 5313 | -1.0634 | 0.006113 |
| FANCC | 2176 | -1.0588 | 0.000925 |
| FDPS | 2224 | -1.0496 | 0.007277 |
| CLRN3 | 119467 | -1.0372 | 0.005085 |
| CES5A | 221223 | -1.0372 | 0.005753 |
| NOL4 | 8715 | -1.037 | 0.001982 |
| GLYCTK | 132158 | -1.0362 | 0.001118 |
| MAP2K6 | 5608 | -1.0291 | 0.000354 |
| RDH16 | 8608 | -1.0241 | 0.024292 |
| EBP | 10682 | -1.0198 | 0.003461 |
| IGFALS | 3483 | -1.0198 | 0.006785 |
| NYNRIN | 57523 | -1.0147 | 0.012424 |
| CYP4A22 | 284541 | -1.0141 | 0.025668 |
| GBP7 | 388646 | -1.0134 | 0.001349 |
| MIRLET7G | 406890 | -1.0105 | 0.00083 |
| NEDD4L | 23327 | 1.0006 | 0.00112 |
| CD84 | 8832 | 1.001 | 0.000289 |
| PIK3IP1 | 113791 | 1.0027 | 0.00159 |
| CD44 | 960 | 1.0134 | 0.00078 |
| SLC25A33 | 84275 | 1.014 | 0.001181 |
| FBN1 | 2200 | 1.0186 | 0.046706 |
| DGKH | 160851 | 1.0202 | 0.005116 |
| LOXL4 | 84171 | 1.0209 | 0.021489 |
| ITK | 3702 | 1.0252 | 0.009918 |
| CPS1-IT1 | 29034 | 1.0257 | 0.011901 |
| SNORA70B | 1E+08 | 1.0279 | 0.000241 |
| FAR2 | 55711 | 1.0295 | 0.014867 |
| GLIPR1 | 11010 | 1.0328 | 0.011635 |
| ABCA9 | 10350 | 1.034 | 0.000335 |
| PLA2G7 | 7941 | 1.0425 | 0.002119 |
| IL32 | 9235 | 1.0438 | 0.007471 |
| LXN | 56925 | 1.0461 | 0.046786 |
| SLAMF8 | 56833 | 1.0484 | 0.004203 |
| PLTP | 5360 | 1.0488 | 0.002424 |
| ANXA1 | 301 | 1.052 | 0.028271 |
| PLXDC2 | 84898 | 1.0566 | 0.013816 |
| GPRIN3 | 285513 | 1.0572 | 0.018161 |
| CD52 | 1043 | 1.0671 | 0.005474 |
| ARNTL | 406 | 1.0816 | 0.005251 |
| KLF7 | 8609 | 1.0855 | 0.000686 |
| SRPX2 | 27286 | 1.0881 | 0.027571 |
| CBLB | 868 | 1.0915 | 0.000207 |
| RBPMS | 11030 | 1.0918 | 4.70E-05 |
| EVI2B | 2124 | 1.0944 | 0.000716 |
| SCARNA5 | 677775 | 1.0984 | 0.002526 |
| PLXNC1 | 10154 | 1.1015 | 0.012348 |
| LOX | 4015 | 1.1054 | 0.009668 |
| ENTPD1 | 953 | 1.1073 | 0.009602 |
| FMN1 | 342184 | 1.1075 | 0.001028 |
| SLCO2A1 | 6578 | 1.1076 | 0.040404 |
| SNORA65 | 26783 | 1.1098 | 0.003219 |
| RNASE6 | 6039 | 1.1111 | 0.005351 |
| MID1 | 4281 | 1.1139 | 0.00067 |
| MYOF | 26509 | 1.115 | 0.029863 |
| C3AR1 | 719 | 1.1262 | 0.00017 |
| SLC22A15 | 55356 | 1.1268 | 0.02842 |
| SORT1 | 6272 | 1.1435 | 0.045797 |
| RPGR | 6103 | 1.1609 | 0.00243 |
| SNORA46 | 677827 | 1.1643 | 0.000172 |
| TNFSF8 | 944 | 1.1697 | 0.002708 |
| TYROBP | 7305 | 1.1717 | 7.72E-05 |
| CCL18 | 6362 | 1.1741 | 0.021878 |
| LEF1 | 51176 | 1.1803 | 0.012717 |
| ADAM28 | 10863 | 1.1884 | 0.011802 |
| PAPPA2 | 60676 | 1.19 | 0.0279 |
| NPAS2 | 4862 | 1.1952 | 0.005515 |
| ANXA2P2 | 304 | 1.201 | 0.005366 |
| CD53 | 963 | 1.2016 | 0.001012 |
| SRGN | 5552 | 1.2086 | 7.99E-05 |
| IL1RL1 | 9173 | 1.2119 | 0.030484 |
| SPRY1 | 10252 | 1.2169 | 0.024292 |
| FLVCR2 | 55640 | 1.2219 | 0.000224 |
| LAPTM5 | 7805 | 1.2228 | 0.000177 |
| ALOX5AP | 241 | 1.2228 | 0.006161 |
| IRAK3 | 11213 | 1.2253 | 0.000865 |
| GREM1 | 26585 | 1.2258 | 0.043506 |
| THY1 | 7070 | 1.2377 | 0.042338 |
| LURAP1L | 286343 | 1.2421 | 0.004958 |
| KCNJ16 | 3773 | 1.2517 | 0.03279 |
| PLP2 | 5355 | 1.2586 | 0.006426 |
| RGS2 | 5997 | 1.2639 | 0.007618 |
| CPA3 | 1359 | 1.2757 | 0.049014 |
| BICC1 | 80114 | 1.2769 | 0.040467 |
| RAB31 | 11031 | 1.307 | 0.000665 |
| SLA | 6503 | 1.3092 | 0.000303 |
| CXCL8 | 3576 | 1.3115 | 0.001233 |
| ERV3-1 | 2086 | 1.327 | 0.000217 |
| EVA1C | 59271 | 1.3331 | 0.000865 |
| CHST9 | 83539 | 1.3343 | 0.006226 |
| ANXA2 | 302 | 1.335 | 0.004283 |
| CCL20 | 6364 | 1.347 | 0.045071 |
| TIMP1 | 7076 | 1.3597 | 0.002876 |
| TSC22D3 | 1831 | 1.3608 | 0.000248 |
| APOLD1 | 81575 | 1.388 | 0.00734 |
| GPNMB | 10457 | 1.39 | 0.007343 |
| PRDM1 | 639 | 1.3906 | 0.000343 |
| SLC1A3 | 6507 | 1.3981 | 0.000916 |
| SNAI2 | 6591 | 1.4012 | 0.000506 |
| SLAMF6 | 114836 | 1.4225 | 0.000827 |
| CAP2 | 10486 | 1.4225 | 0.001523 |
| ZBTB16 | 7704 | 1.4411 | 0.001336 |
| ANKRD1 | 27063 | 1.4568 | 0.007018 |
| SCARNA1 | 677774 | 1.4597 | 0.009257 |
| NRG1 | 3084 | 1.4754 | 0.000485 |
| GPR34 | 2857 | 1.4955 | 4.70E-05 |
| UBD | 10537 | 1.4978 | 0.018077 |
| SNORD56B | 319139 | 1.5234 | 1.13E-05 |
| SNORA16B | 692157 | 1.5281 | 9.88E-05 |
| CYTIP | 9595 | 1.5314 | 0.00014 |
| MYOM1 | 8736 | 1.5486 | 0.004723 |
| RNU4-2 | 26834 | 1.6466 | 0.018079 |
| MOXD1 | 26002 | 1.6469 | 0.03157 |
| CD69 | 969 | 1.669 | 0.004132 |
| NEDD9 | 4739 | 1.6819 | 0.000177 |
| CCL21 | 6366 | 1.7056 | 0.005462 |
| SNORA20 | 677806 | 1.7252 | 0.044103 |
| DTNA | 1837 | 1.7496 | 0.004092 |
| IL7R | 3575 | 1.768 | 0.005724 |
| MGP | 4256 | 1.7919 | 0.03279 |
| UPP2 | 151531 | 1.8834 | 0.001289 |
| VCAN | 1462 | 1.9533 | 0.025825 |
| SPP1 | 6696 | 2.0299 | 0.019691 |
| SCN7A | 6332 | 2.1042 | 0.014747 |
| EFEMP1 | 2202 | 2.1175 | 0.023629 |
| CXCR4 | 7852 | 2.1319 | 0.000573 |
| RGS1 | 5996 | 2.3372 | 8.50E-05 |
| STMN2 | 11075 | 2.557 | 0.015541 |
| MMP7 | 4316 | 2.797 | 0.00552 |
